# Device Simulation of Highly Stable and 29% Efficient ${FA}_{0.75}{MA}_{0.25}{Sn}_{0.95}{Ge}_{0.05}I_{3}$-Based Perovskite Solar Cell

**DOI:** 10.3390/nano13091537

**Published:** 2023-05-03

**Authors:** Hussein Sabbah, Zaher Abdel Baki

**Affiliations:** College of Engineering and Technology, American University of the Middle East, Egaila 54200, Kuwait; zaher.abdelbaki@aum.edu.kw

**Keywords:** solar cell, photovoltaics, thin films, SCAPS simulation, lead-free perovskite, power conversion efficiency, Sn:Ge perovskite

## Abstract

A new type of perovskite solar cell based on mixed tin and germanium has the potential to achieve good power conversion efficiency and extreme air stability. However, improving its efficiency is crucial for practical application in solar cells. This paper presents a quantitative analysis of lead-free FA_0.75_MA_0.25_Sn_0.95_Ge_0.05_I_3_ using a solar cell capacitance simulator to optimize its structure. Various electron transport layer materials were thoroughly investigated to enhance efficiency. The study considered the impact of energy level alignment between the absorber and electron transport layer interface, thickness and doping concentration of the electron transport layer, thickness and defect density of the absorber, and the rear metal work function. The optimized structures included poly (3,4-ethylenedioxythiophene)polystyrene sulfonate (PEDOT:PSS) as the hole transport layer and either zinc oxide (ZnO) or zinc magnesium oxide (Zn_0.7_Mg_0.3_O) as the electron transport layer. The power conversion efficiency obtained was 29%, which was over three times higher than the initial structure. Performing numerical simulations on FA_0.75_MA_0.25_Sn_0.95_Ge_0.05_I_3_ can significantly enhance the likelihood of its commercialization. The optimized values resulting from the conducted parametric study are as follows: a short-circuit current density of 30.13 mA·cm^−2^), an open-circuit voltage of 1.08 V, a fill factor of 86.56%, and a power conversion efficiency of 28.31% for the intended solar cell.

## 1. Introduction

Renewable energy has become a crucial aspect of global energy production due to the increasing demand for clean and sustainable sources of energy. Research into solar cell technology is highly appealing and holds great promise, since sunlight is an unlimited and free resource that is both fundamentally renewable and environmentally friendly in contrast to finite fossil fuels [1,2]. In this context, the use of metal halide perovskites (MHP) in photovoltaics has seen tremendous growth in the past 10 years, with the recent power conversion efficiency (PCE) reaching above 25% [3,4,5]. Despite this progress, the challenge lies in the fact that all the current MHP used to achieve high PCE contain lead, making it doubtful that this technology will be adopted on a large scale, especially in Europe, where strict regulations against the use of lead in electronics have been put in place [6].

As a result, there has been a growing interest in recent years to replace the lead component (Pb) in the perovskite formula $APbX_{3}$ with less toxic elements such as tin (Sn), bismuth (Bi), antimony (Sb), copper (Cu), or germanium (Ge) for both photovoltaic applications and crystal formation [7]. Of these alternatives, Sn-based perovskites [8,9] have been the most extensively studied and have demonstrated impressive PCE of up to 13% [10]. Sn-based perovskites have the advantage of smaller optical bandgaps [11,12,13] and greater charge mobility [14] compared to their Pb-based counterparts, making them ideal for single junction solar cells and all-perovskite tandem solar cells. Additionally, Sn is a naturally abundant element that does not present any environmental or health hazards. Perovskite solar cells (PSCs) based on Sn are widely recognized for their instability when exposed to the air due to the tendency of Sn to oxidize from a +2 to +4 state, which results in the creation of oxygen vacancies that can act as traps [2,15,16].

Another potential replacement for lead is Ge, a group 14 elements such as Sn and Pb. Germanium is a strong candidate for perovskite solar cells, as it has a higher electronegativity and more covalent character than lead [13]. Despite numerous theoretical studies suggesting the potential of germanium halide perovskites for solar cell applications [17,18,19,20], they have only rarely been studied experimentally due to their mercurial character in a +2 oxidation state [21]. To date, the PCE of Ge-based PSC is still below 5% due to factors such as a smaller ionic radius, limited solubility in polar solvents, and a relatively wide bandgap (>1.6 eV) [18,22,23]. Although lead-free perovskites have demonstrated good efficiencies, there remains a persistent need to improve their stability and effectiveness [24,25,26,27], as their power conversion efficiency (PCE) values still fall considerably below the Shockley–Queisser efficiency limit of 33.7% [28] for a single junction. It is important to note that this limit has been exceeded by using nanoscale metallization in perovskite solar cells [28,29,30,31]. However, although metallization represents progress in perovskite technology, it is unlikely to significantly change the market situation for these cells. The primary challenge for perovskite cells is their poor durability and rapid degradation in the presence of oxygen and atmospheric water. To address the issue of oxidation in PSC, various techniques have been explored [16,32,33,34,35], including changing the electronic structure of the perovskite material, the encapsulation of PSC, incorporating hydrogen bonding, and applying a hydrophobic layer, among others [32,36]. 

One of the techniques for enhancing the performance of PSC involves mixing cations in tin–germanium Sn:Ge-based PSC. This has shown positive results in the literature [17,37,38,39]. By changing the Sn:Ge ratio, researchers have been able to achieve a lower bandgap and improved stability [40]. According to a study by Ito et al. [38], the efficiency of pure Sn-based perovskites improved from 3.31% to 4.48% (and even further to 6.90% after 72 h) when 5% germanium was doped into the material. The measurement was taken in the air without encapsulation. The addition of germanium is believed to have increased the stability of the perovskite structure while decreasing the trap density. This trend was also observed by Ng et al. [39], as they recorded the highest PCE (7.9%) of Sn:Ge-based PSC to date. The efficiency of the Sn:Ge-based PSC is significantly lower than that of the Pb-based version due to low $V_{oc}$ and $J_{sc}$. This is likely caused by poor absorption at the UV range, as noted in prior research [41,42,43], and issues with the energy level alignment at the interface electron transport layer (ETL)/perovskite [44,45]. 

Further improvement in the PCE is still required, as the experimental outcomes have yet to reach the 25% PCE recorded by lead-based perovskite solar cells [44]. Studying the properties of the materials used in solar cells and controlling them through reliable simulation software can result in the creation of solar cells that are both highly efficient and cost-effective. PSC simulation is an interesting and straightforward process that can be carried out using various trustworthy programs such as SCAPS, AFORS-HET, Sentarus, and Silvaco [43,46,47,48,49].

In this contribution, we aim to enhance the efficiency of ${FA}_{0.75}{MA}_{0.25}{Sn}_{0.95}{Ge}_{0.05}I_{3}$-based solar cells by utilizing SCAPS software, developed by Gent University [50]. This particular perovskite has demonstrated a good PCE of 7.9% and impressive air stability in previous experiments [38,39]. This study presents a simple yet comprehensive simulation of the ${FA}_{0.75}{MA}_{0.25}{Sn}_{0.95}{Ge}_{0.05}I_{3}$-based PSC with a conventional (n-i-p) planar structure, which has not been previously conducted. The simulation mainly focuses on the use of metal oxide transport layers, particularly ZnO and ${Zn}_{0.7}{Mg}_{0.3}O$. These materials have suitable electronic energies, high transparency, and uniform substrate coverage, making them excellent candidates for an ETL in the low-cost and large-scale production of lead-free PSC [51,52]. 

To attain maximum efficiency, an optimization process is carried out. First, a range of ETL materials are evaluated, and the most suitable ones are chosen. Then, their thicknesses and doping concentrations are optimized. Following this, the thickness of the perovskite absorber layer and its defect density are optimized. The effect of the rear metal work function on the photovoltaic performance of the device is analyzed subsequently. Finally, the results of the optimized structure are presented, demonstrating an improvement in efficiency of around 29%.

## 2. Materials and Methods

The design and performance analysis of a solar cell were conducted using the SCAPS-1D software program. This numerical simulation tool was developed by researchers in the Department of Electronics and Information Systems (ELIS) at the University of Gent in Belgium [50]. The simulation program solves Poisson’s equation and the continuity equation for free electrons and holes in the conduction and valence bands. It enables the computation and observation of various electrical properties and parameters, such as the current density–voltage characteristics (J–V curve), the energy band structure of the heterojunction, quantum efficiency (QE), open circuit voltage ($V_{oc}$), short circuit current (Jsc), current density, power conversion efficiency (PCE), and fill factor (FF), among others. All simulations were conducted at a temperature of 300 K under the standard illumination of $1000\;{W/m}^{2}$ and an air mass of AM 1.5 G. The absorber layer was sandwiched between the hole transport layer (HTL) and ETL layers.

**Figure 1:**
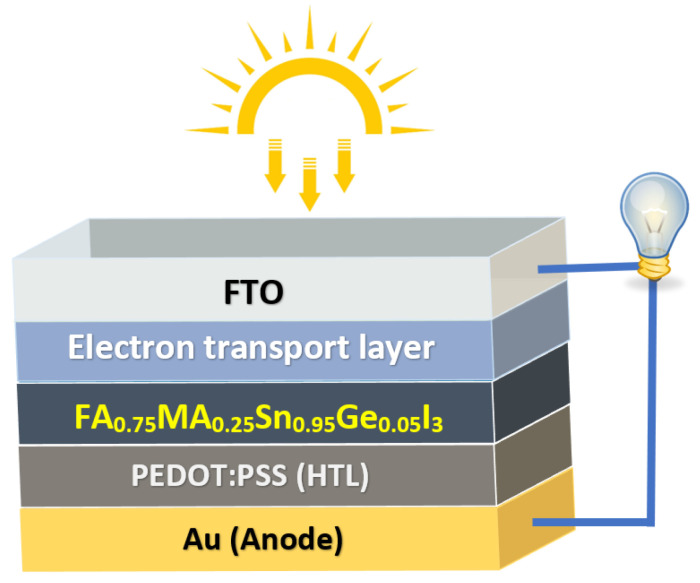
Schematic diagram of a PSC based on mixed Sn:Ge ${FA}_{0.75}{MA}_{0.25}{Sn}_{0.95}{Ge}_{0.05}I_{3}$.

Figure 1 illustrates the proposed PSC structure with $fluorine-dopedtinoxide(FTO)/ETL/{FA}_{0.75}{MA}_{0.25}{Sn}_{0.95}{Ge}_{0.05}I_{3}/\left(PEDOT:PSS\right)/Gold(Au)$. The solar cell has a conventional structure (n-i-p), meaning that light enters the cell from the ETL side, with FTO acting as the front contact and Au as the back contact. For the HTL, PEDOT:PSS is used in every structure. However, instead of using organic ETLs fullerene (C60) and [6,6]-phenyl-C(61)-butyric acid methyl ester (PCBM), as in the experimental work, this study investigated two ideal ETLs, ZnO and ${Zn}_{0.7}{Mg}_{0.3}O$, as well as the conventional and extensively studied ETL titanium dioxide (${TiO}_{2}$). The photovoltaic performance of the cell was compared using three different metal oxides (${TiO}_{2}$, ZnO, and ${Zn}_{0.7}{Mg}_{0.3}O$), alternately used as the ETL layer with the two organic ETLs C60 and PCBM.

**Figure 2:**
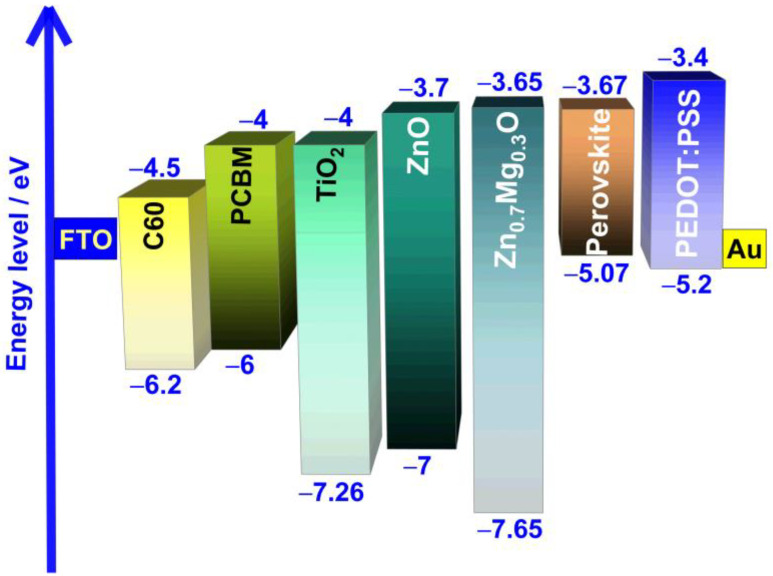
Band alignment between the ETL materials and ${FA}_{0.75}{MA}_{0.25}{Sn}_{0.95}{Ge}_{0.05}I_{3}$ perovskite.

The energy level diagram of the perovskite with two organic ETLs and three inorganic metal oxide ETLs, along with other layers, is depicted in Figure 2.

Table 1, Table 2 and Table 3 provide a summary of the device and material parameters that were taken from theories, experiments, and the literature. The parameters listed in the table were considered while creating the initial setup for the simulation process. Various properties, such as the thickness and doping concentration of the ETL, the thickness and defect density of the absorber layer, and the rear metal work function, were adjusted to achieve the best possible outcome and to examine their impact on the device’s performance.

Aside from the earlier study that involved altering the ETL materials, various parameters, including the thickness and doping concentration of the ideal ETL layers and absorber, the defect density of the perovskite layer, and the rear metal work function of the cells, were adjusted to evaluate their effects on the device’s performance. The goal was to achieve the most effective cell structure through these modifications.

## 3. Results and Discussion

In this section, the study’s findings are presented, which started by examining the impact of different ETLs on the solar cell performance. After identifying the best structures based on this analysis, the research then explored various factors. These included optimizing the thickness and doping concentration of the ideal ETL, refining the absorber thickness, assessing the effect of the absorber layer’s defect density, and investigating how the solar cell’s performance was affected by the rear metal work function. 

### 3.1. Impact of ETL Material on Solar Cell Performance

**Figure 3:**
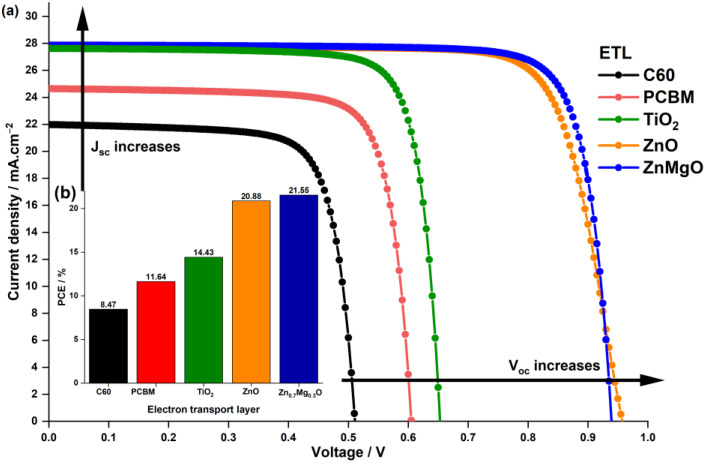
Impact of the ETL material on (**a**) the current density–voltage characteristics and (**b**) the PCE of the PSC.

In planar PSC, the interface between the ETL and perovskite absorber layer plays a vital role in determining their overall performance [45]. To ensure high-quality ETL, several properties must be considered [44]. Firstly, the ETL should possess a suitable lowest unoccupied molecular orbital (LUMO) energy level that matches the conduction band energy of perovskite materials. Secondly, it should have high electron mobility and photochemical stability under solar irradiation. Lastly, it should be optically transparent to ensure maximum light absorption by the perovskite layer in the n-i-p PSC. Therefore, various ETLs, including, C60, PCBM, ${TiO}_{2}$, ZnO, and ${Zn}_{0.7}{Mg}_{0.3}O,$ are being examined to investigate how device performances differ with the uses of different ETLs. The electrical and optical parameters of the ETLs are listed in Table 2. Figure 3 depicts both the impact of the ETL material on the current density–voltage characteristics and its effect on the PCE. 

Table 4 lists the solar cell performance metrics, as well as the conduction band offset (CBO) values, for the simulated devices with five different ETLs. CBO refers to the difference in electron affinity between the absorber and the ETL (Equation (1)).

(1) $$CBO=\chi_{absorber}-\chi_{ETL}$$

Figure 3 clearly shows that PSC with the organic ETL materials C60 and PCBM have low performance, yielding PCE values below 12%. In contrast, all the structures with inorganic ETL materials produce PCE values above 14%. Among the structures simulated, those incorporating ZnO ETL and ${Zn}_{0.7}{Mg}_{0.3}O$ ETL materials are the most efficient, achieving PCE values of 20.88% and 21.55%, respectively. 

Devices incorporating organic ETLs and ${TiO}_{2}$ ETL exhibit significantly lower PCEs than PSCs utilizing ZnO ETL and ZnMgO ETL, primarily due to their lower $V_{oc}$. The low $V_{oc}$ is likely attributable to the band alignment present within their structure. Table 4 reveals that, as the CBO becomes increasingly negative, the $V_{oc}$ value decreases correspondingly. PSCs incorporating ZnO ETL or ${Zn}_{0.7}{Mg}_{0.3}O$ ETL, on the other hand, exhibit CBO values close to zero or even positive, which accounts for their comparatively higher $V_{oc}$ values. When the conduction band minimum (CBM) of the ETL is located below that of the absorber, it results in a negative CBO, and a cliff-like structure forms at the heterojunction ETL/absorber. In solar cells, this cliff structure is detrimental, since it promotes the accumulation of electrons and holes near the interface following charge separation, leading to greater charge recombination via the interface’s deep-level defects, which results in lower $V_{oc}$.

Alongside the band alignment within the structure of the PSC, another factor that could contribute to the difference in behavior between the devices is the ETL’s bandgap. A suitable ETL material must possess optical transparency to ensure maximum light absorption by the perovskite layer in the n-i-p PSC, which leads to the generation of more electrons and ultimately results in a higher $J_{sc}$ achieved by the cell. This claim is supported by Figure 3 and Table 4. PSCs with organic ETLs, which have bandgap values of 1.7 eV and 2 eV, respectively, demonstrate the lowest values of $J_{sc}$, specifically $21.97\;{mA\cdot cm}^{-2}$ and $24.64\;{mA\cdot cm}^{-2}$. Conversely, PSCs with inorganic metal oxide ETLs score the highest values of $J_{sc}$, above $27\;{mA\cdot cm}^{-2}$, as they have bandgap values above 2.8 eV. 

**Figure 4:**
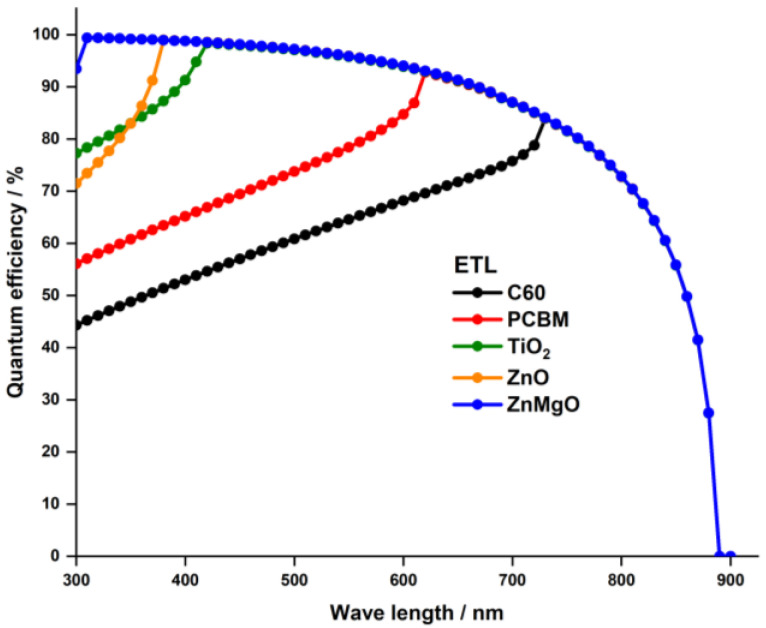
Quantum efficiency for the simulated devices with different ETL materials.

Figure 4 endorses this observation, illustrating the quantum efficiency of PSCs with various ETLs. PSCs with organic ETLs have a notably low quantum efficiency, particularly for wavelengths below 700 nm, attributable to their below −2 eV bandgap values. On the other hand, PSCs with inorganic metal oxide ETLs exhibit the highest quantum efficiency, since their bandgap values exceed 2.8 eV.

The results presented in this section demonstrate that ZnO and ${Zn}_{0.7}{Mg}_{0.3}O$ outperform the other tested ETLs. Consequently, the study investigates how the thickness and doping concentration of these ETL materials affect the performances of solar cell devices.

#### 3.1.1. Impact of ETL Thickness

**Figure 5:**
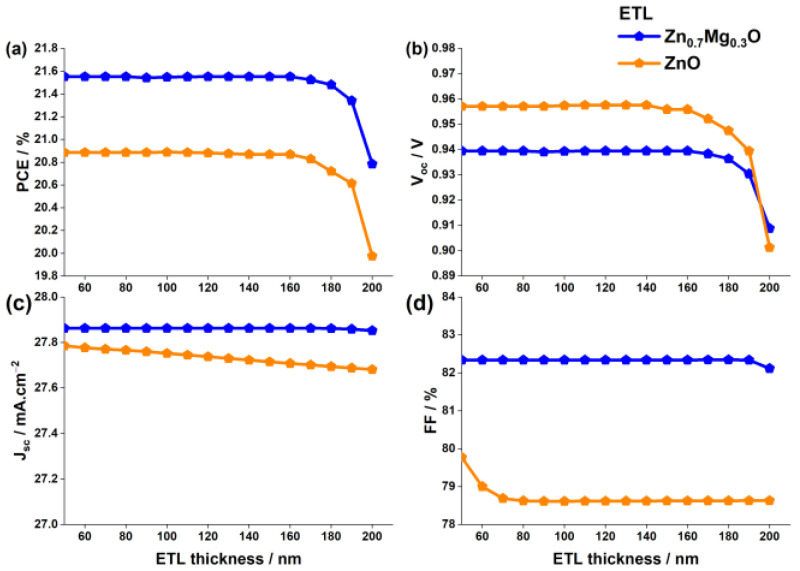
Variations of (**a**) PCE, (**b**) $V_{oc}$, (**c**) $J_{sc}$, and (**d**) FF as a function of the ETL thickness for the two ETL materials ZnO and ${Zn}_{0.7}{Mg}_{0.3}O$.

In the previous analysis, we compared the ETL materials while keeping the layer thickness constant at 50 nm. However, in this section, we study the impact of varying the thickness of the ETL on the solar cell’s performance. We varied the ETL thickness from 50 nm to 200 nm and analyzed the photovoltaic performances. Figure 5 shows the results as a function of the ETL thickness. The $V_{oc}$ and PCE of both devices remain constant and independent of an ETL thickness up to around 160 nm. However, they decrease significantly as the thickness increases further. The increase in thickness causes electrons to travel a longer distance to reach the top electrode, resulting in a higher likelihood of electron recombination with minority carriers (holes). This, in turn, causes the $V_{oc}$ to decline sharply. Additionally, the cell with a ZnO ETL shows a noticeable decrease in $J_{sc}$, likely due to a decrease in light transmittance through the ZnO layer.

**Figure 6:**
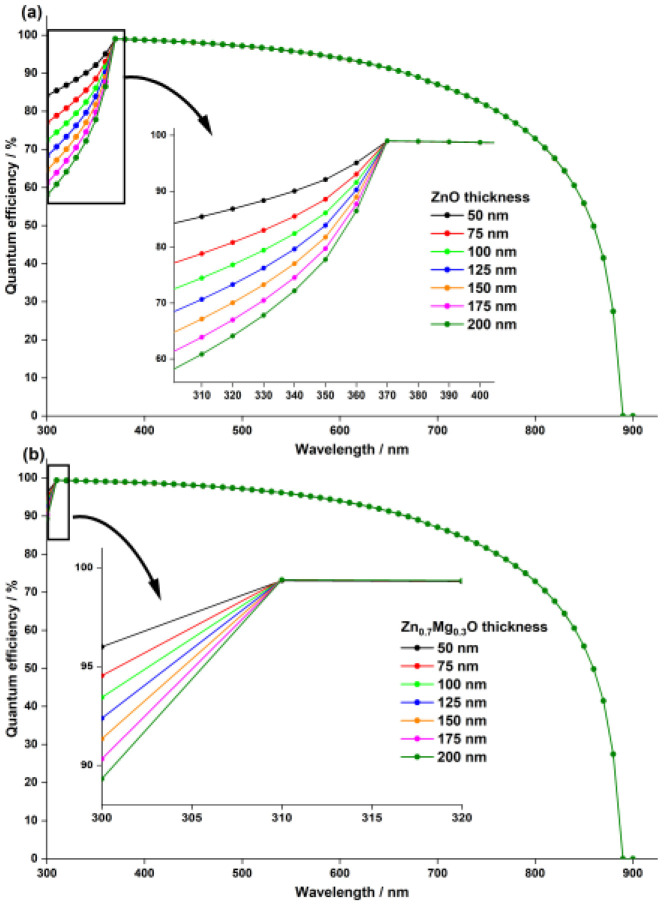
Effect of ETL thickness on the quantum efficiency of the PSC with two different ETL materials: (**a**) ZnO and (**b**)  ${Zn}_{0.7}{Mg}_{0.3}O$.

This decrease in light transmittance is confirmed by Figure 6, which shows the quantum efficiency of the cells as a function of the ETL thickness. The QE and the $J_{sc}$ of the cell with a Zn_0.7Mg_0.3O ETL remain unchanged due to the wide bandgap of ${Zn}_{0.7}{Mg}_{0.3}O$ (4.1 eV).

It is clear that the photovoltaic parameters deteriorate as the ETL thickness increases, resulting in a decrease in PCE for both ETLs. This is caused by inefficient charge carrier transport to the electrodes, an increase in series resistance that reduces the fill factor FF, and a higher probability of recombination as the ETL thickness increases. Therefore, for the remainder of this study, a thickness of 50 nm is adopted. Any thinner layer may not fully cover the perovskite layer, causing direct contact between FTO and perovskite, which leads to carrier recombination and reduced hole-blocking efficiency [63].

#### 3.1.2. Impact of ETL Doping Concentration

**Figure 7:**
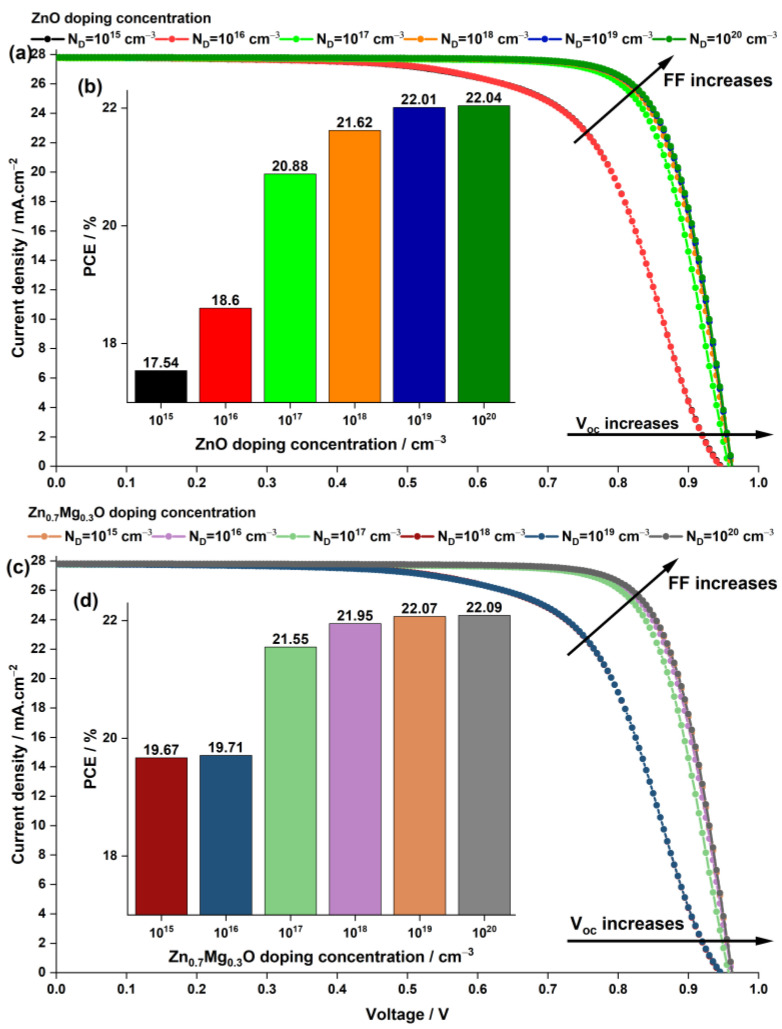
Effect of the doping concentration of the ETL ZnO on (**a**) the current density–voltage characteristics of the PSC and (**b**) the PCE, and the effect of the doping concentration of ETLZn_0.7_Mg_0.3_O on (**c**) the current density–voltage characteristics of the PSC and (**d**) the PCE.

In addition to identifying the most suitable ETL materials, namely ZnO and ${Zn}_{0.7}{Mg}_{0.3}O$, and optimizing their thickness at 50 nm, it is important to consider the impact of the doping concentration $N_{D}$ on the photovoltaic parameters of PSCs. In the previous sections, a fixed doping concentration of $N_{D}=1\times 10^{17}\;{cm}^{-3}$ was used for all materials tested. However, this section presents a study on the effect of varying the $N_{D}$ from $1\times 10^{15}\;{cm}^{-3}$ to $1\times 10^{20}\;{cm}^{-3}$ for the current density–voltage characteristics and power conversion efficiency (PCE), as shown in Figure 7.

The results shown in Figure 7 clearly demonstrate that increasing the doping concentration in the ETL significantly enhances the $V_{oc}$ and FF of the cells, resulting in a higher PCE. Notably, the $J_{sc}$ of both devices remains almost constant, as it has already reached a high value of approximately $28\;{mA\cdot cm}^{-2}$, and the effect of doping on $J_{sc}$ is negligible.

Both devices achieved a peak PCE of 22% at doping concentrations of $1\times 10^{19}\;{cm}^{-3}$ and $1\times 10^{20}\;{cm}^{-3},$ with little difference between them. The improvement in $V_{oc}$ and FF can be attributed to the effect of doping on the energy level alignment between the ETL and the perovskite layer, which enhances the charge transport properties and reduces the recombination losses. 

Although increasing the doping concentration of the ETL improves the $V_{oc}$, FF, and overall efficiency of the PSC, the optimal doping concentration has been determined to be $1\times 10^{19}\;{cm}^{-3}$ due to practical manufacturing challenges. Higher doping concentrations are difficult to achieve practically and could potentially create deep Coulomb traps, which may adversely affect carrier mobility [64].

### 3.2. Effect of the Perovskite Layer on the Solar Cell Performance

In addition to the crucial role of ETL materials in improving the PSC performance, the absorber layer also has a significant impact on the efficiency of the solar cell. This section will examine how the absorber material ${FA}_{0.75}{MA}_{0.25}{Sn}_{0.95}{Ge}_{0.05}I_{3}$ affects the solar cell performance, with a specific focus on the thickness and defect density of this absorber.

#### 3.2.1. Impact of Absorber Thickness

**Figure 8:**
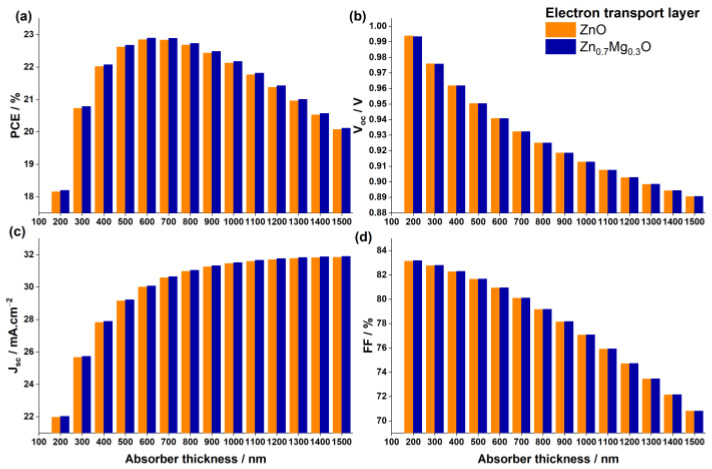
Impact of the absorber thickness on (**a**) PCE, (**b**) $V_{oc}$, (**c**) $J_{sc}$, and (**d**) FF for PSCs with the two ETL materials ZnO and ${Zn}_{0.7}{Mg}_{0.3}O$.

**Figure 9:**
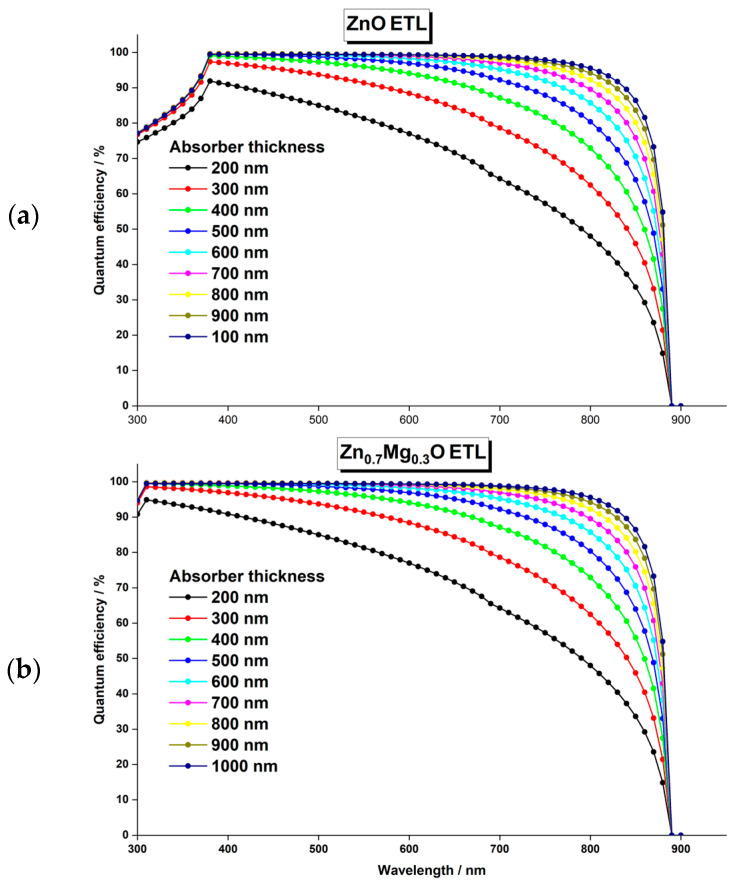
Impact of the absorber thickness on the quantum efficiency of PSCs with (**a**) ZnO ETL and (**b**) ${Zn}_{0.7}{Mg}_{0.3}O$ ETL.

The thickness of the absorber layer in perovskite solar cells can significantly impact the device’s performance, as it determines the amount of light absorption and the efficiency of the conversion process. It is crucial to maintain an optimal thickness range, because if the absorber layer is too thin, it may not absorb sufficient light to generate enough current. Conversely, if the absorber layer is too thick, the charge carriers generated by the absorbed light may struggle to travel through the material and reach the electrodes, leading to lower device efficiency. The previous analyses were performed using a 400 nm thick ${FA}_{0.75}{MA}_{0.25}{Sn}_{0.95}{Ge}_{0.05}I_{3}$. In this section, the impact of the absorber thickness on the solar cell’s performance was studied by varying the thickness from 200 nm to 1500 nm. The results obtained for the photovoltaic outputs are shown in Figure 8, while Figure 9 displays the effect of the absorber thickness on the quantum efficiency with respect to the wavelengths of the light.

By observing Figure 8 and Figure 9, it is evident that the behavior of all photovoltaic parameters and the quantum efficiency as a function the of absorber thickness is similar for both devices, with ZnO ETL and ${Zn}_{0.7}{Mg}_{0.3}O$ ETL. The PCE of both devices steadily increases with the increasing thickness until it reaches a maximum value of 22.9% at 600 nm, beyond which it gradually decreases. This can be attributed to the opposing trends of the $V_{oc}$ and the $J_{sc}$, which have the most significant impact on the PCE.

Firstly, a considerable increase in $J_{sc}$ was observed in both devices by increasing the thickness of the absorber layer, but it reached saturation at 700 nm. Beyond this point, the effect of the absorber layer thickness became insignificant. This substantial enhancement of $J_{sc}$ is attributed to the generation of additional electron–hole pairs in the perovskite, which occurred as a result of increased light absorption resulting from the thicker absorber layer. The increase in $J_{sc}$ is supported by the higher QE of the device at larger thicknesses, as shown in Figure 9.

In contrast, the open-circuit voltage $V_{oc}$ decreases as the absorber layer thickness increases. While a thicker layer allows for more photons to be absorbed and more electron–hole pairs to be generated, it also leads to a high density of defects that act as recombination centers. As a result, the lifetime of electron–hole pairs is reduced, and more pairs recombine before reaching the electrodes, causing a decline in the $V_{oc}$. In addition, the series resistance of the device increases with thicker absorber layers, further lowering the $V_{oc}$ and the FF. Consequently, increasing the absorber layer thickness beyond 600 nm results in diminishing returns and decreases the overall efficiency of the solar cell.

#### 3.2.2. Impact of Absorber Defect Density $N_{t}$

Although adjusting the thickness of the absorber has improved the efficiency of solar cells, further enhancements in the solar cell performance can be achieved by considering the defect density of the perovskite layer as an additional influential parameter. 

**Figure 10:**
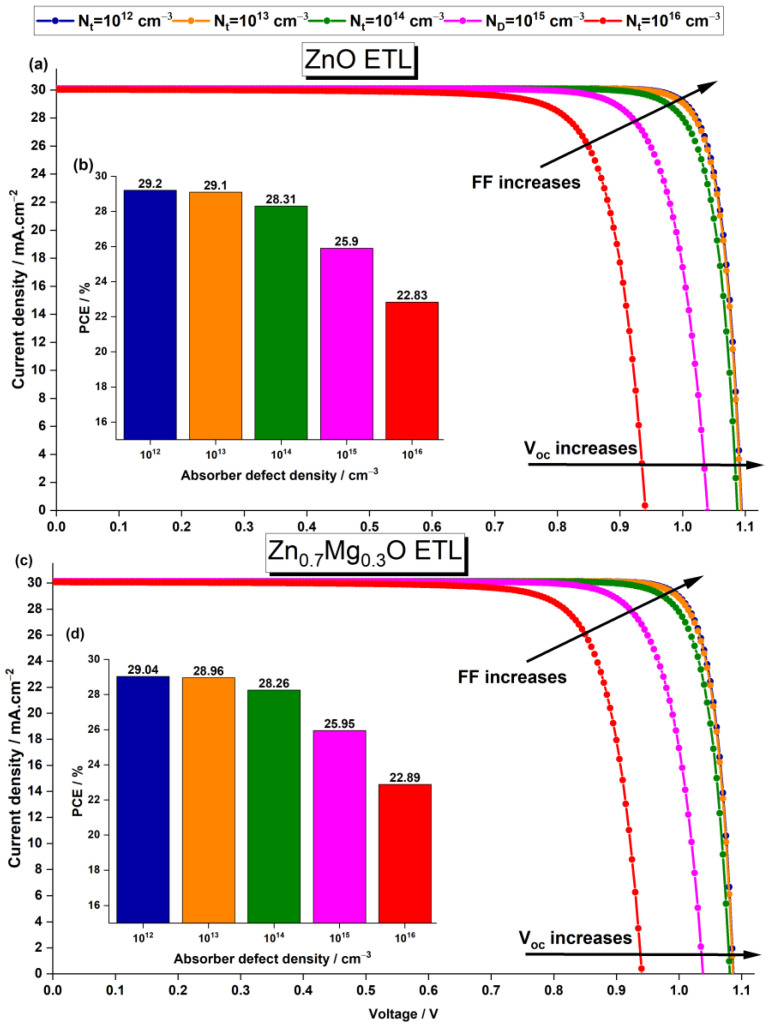
Effect of the absorber defect density on the current density–voltage characteristics and the PCE of the PSC with ZnO ETL (**a**,**b**) and ${Zn}_{0.7}{Mg}_{0.3}O$ ETL (**c**,**d**), respectively.

The initial defect density $N_{t}$ of the absorber was set at $1\times 10^{16}\;{cm}^{-3}$, which is the same as the value obtained in the experiment conducted by Ng et al. [39] on ${FA}_{0.75}{MA}_{0.25}{Sn}_{0.95}{Ge}_{0.05}I_{3}$. Recent experimental studies by Zheng et al. [65] and Chen et al. [66] showed that the defect density in perovskite can be as low as $1\times 10^{11}\;{cm}^{-3}$ and $1\times 10^{12}\;{cm}^{-3}$, respectively. In our simulation study, we varied the defect density between $1\times 10^{12}\;{cm}^{-3}$ and $1\times 10^{16}\;{cm}^{-3}$ and plotted the changes in the photovoltaic properties with $N_{t}$ for devices using ZnO ETL and ${Zn}_{0.7}{Mg}_{0.3}O$ ETL in Figure 10.

The $J_{sc}$ of both devices remain constant, while $V_{oc}$ and FF are greatly increased when the defect density in perovskite is reduced, resulting in a significant enhancement of the PCE. When the defect density reaches a low level of $1\times 10^{13}\;{cm}^{-3}$, both cells show a significant improvement in performance. The cells with ZnO ETL and ${Zn}_{0.7}{Mg}_{0.3}O$ ETL exhibit a $J_{sc}$ of $30.11\;mA\cdot {cm}^{-2}$ and $30.11\;mA\cdot {cm}^{-2}$, $V_{oc}$ of 1.087 V and 1.087 V, FF of 86.46% and 86.74%, and PCE of 28.31% and 28.26%, respectively. However, further reducing the $N_{t}$ from $1\times 10^{13}\;{cm}^{-3}$ to $1\times 10^{12}\;{cm}^{-3}$ only leads to a slight improvement in the cell performance. Hence, an absorber defect density $N_{t}=1\times 10^{13}\;{cm}^{-3}$ is adopted for the rest of this study. 

**Figure 11:**
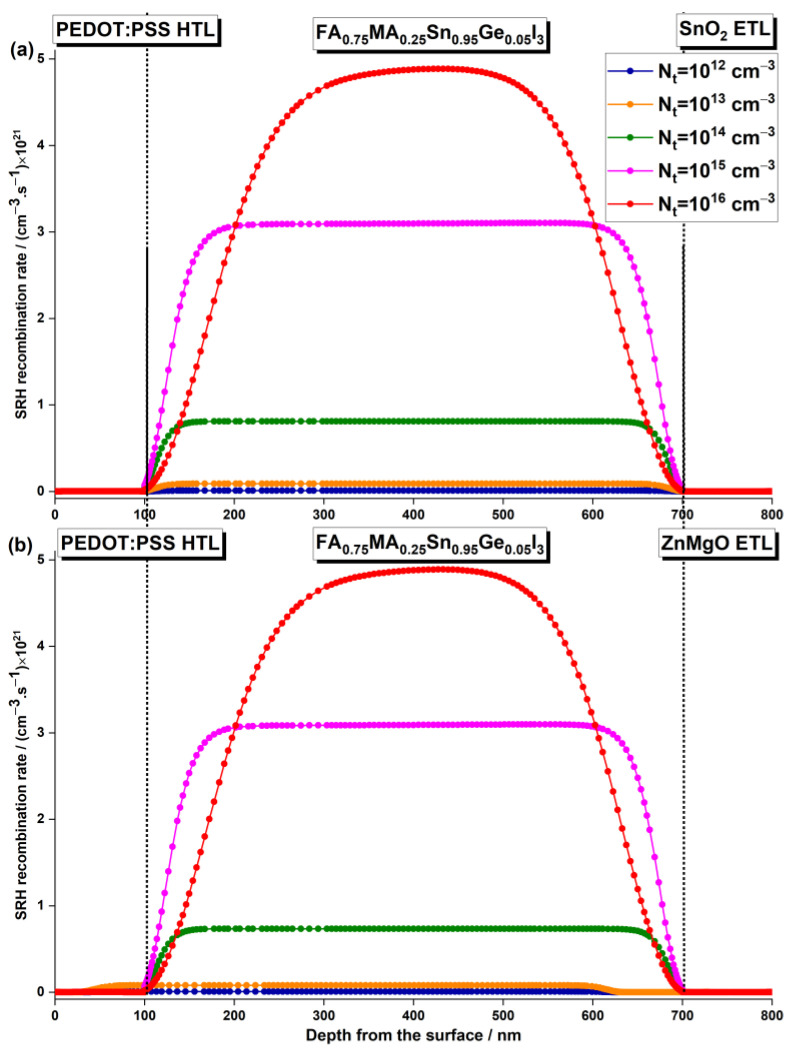
Effect of the absorber layer defect density on the recombination rate with the depth from the surface of the PSC with (**a**) ZnO ETL and (b) ${Zn}_{0.7}{Mg}_{0.3}O$ ETL.

The impact of perovskite defect density on the device performance can be explained by the Shockley–Read–Hall (SRH) recombination model [64,67]. To gain a better understanding of this effect, the relationship between the SRH recombination rate and depth from the surface for various defect densities was examined. Figure 11 illustrates these findings. The results indicate that recombination becomes more significant in the light-absorbing layer due to the higher defect density resulting from the low film quality. The defects in the absorber layer act as recombination centers for the electron–hole pairs generated by incident photons, which reduces the efficiency of the device. The defect density in the absorber layer can be influenced by a variety of factors, such as the synthesis process, the perovskite composition, and the deposition method. Therefore, greater efforts should be made to improve the fabrication technique of solar cells.

### 3.3. Impact of Rear Electrode Work Function

The work function of the rear metal in a PSC plays a critical role in determining the energy alignment at the interface between the HTL and the rear contact electrode, which affects the built-in potential $V_{bi}$ and the charge carrier extraction. In this study, the rear electrode in the PSC was initially made of gold (Au), which is a common choice for metal back contact. However, other materials such as aluminum (Al), silver (Ag), chromium (Cr), nickel (Ni), palladium (Pd), and platinum (Pt), with different work functions ranging from 4.2 to 5.7 eV, are also used in PSCs and optoelectronic devices. Table 5 presents the work functions of several chosen metals in the field [68]. 

**Figure 12:**
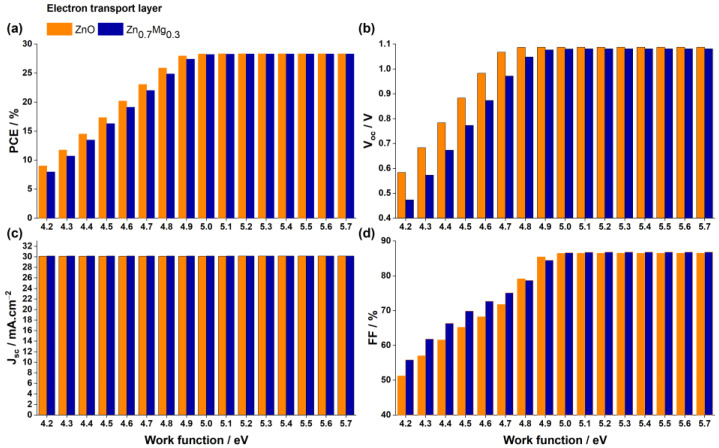
Impact of the work function of the anode on the (**a**) PCE, (**b**) $V_{oc}$, (**c**) $J_{sc}$, and (**d**) FF for PSCs with the two ETL materials ZnO and ${Zn}_{0.7}{Mg}_{0.3}O$.

Our study aimed to examine how the rear metal work function affects the photovoltaic properties of the devices. The results are depicted in Figure 12.

As the work function of the anode increases, both the $V_{oc}$ and FF of the solar cell increase, resulting in greater efficiency, until they reach a maximum and plateau at 5 eV and above. This is because a decrease in the metal work function reduces the built-in electric field in the absorber layer, causing a poor collection of photo-generated carriers and resulting in lower $V_{oc}$ and FF.

In cases where the anode’s work function is lower than that of PEDOT:PSS (5.0 eV) (5.0 eV) [69], with metals such as Ag, Cu, and Au, a rectifying Schottky barrier contact is created at the anode–PEDOT interface. This contact acts as an obstacle to the movement of holes to the anode, thereby decreasing the $V_{oc}$, FF, and PCE, as illustrated in Figure 12. On the other hand, when using an anode made of Au, Ni, Pd, or Pt, which have a higher work function than PEDOT, an ohmic contact is established at the anode–PEDOT:PSS interface. This enables efficient hole transport across the interface, resulting in higher $V_{oc}$, FF, and PCE values in the PSC. Therefore, selecting one of these anodes is crucial when manufacturing the solar cell device.

## 4. Conclusions

Despite conducting extensive experimental studies on ${FA}_{0.75}{MA}_{0.25}{Sn}_{0.95}{Ge}_{0.05}I_{3}$-based PSCs, the highest achieved PCE remained below 8%, which falls short of the desired benchmark for commercial applications. Our work utilized SCAPS-1D software to simulate a conventional (n-i-p) structure and systematically compared the effectiveness of various ETL materials. Specifically, we tested two organic ETLs and three inorganic metal oxide ETLs while keeping the other layers unchanged. Our findings showed that selecting appropriate ETL materials could significantly increase the PCE of the cell to 21%. The ZnO and ${Zn}_{0.7}{Mg}_{0.3}O$ ETLs were found to be the most effective ETL materials due to their excellent band alignments with the absorber and wide bandgaps. The efficiency of the solar cell was further improved by increasing the doping concentration of the ETL and the absorber thickness, reducing the absorber defect density, and selecting gold or any metal with a work function greater than 5.1 eV. These enhancements led to an unprecedented PCE of almost 29%. It is crucial to be cautious while interpreting these findings, because they may not accurately represent the experimental efforts, which were only able to achieve a maximum PCE of less than 8%. Future research should focus on refining the device fabrication techniques, as our novel results could provide a feasible approach to develop cost-effective, highly efficient, and stable ${FA}_{0.75}{MA}_{0.25}{Sn}_{0.95}{Ge}_{0.05}I_{3}$-based PSCs.

## Figures and Tables

**Table 1:** Electrical and optical properties employed in the simulation of ${FA}_{0.75}{MA}_{0.25}{Sn}_{0.95}{Ge}_{0.05}I_{3}$-based PSC.

Parameters	C60 (ETL) [53,54,55,56]	${FA}_{0.75}{MA}_{0.25}{Sn}_{0.95}{Ge}_{0.05}I_{3}$ (Aborsober) [38,39,57]	PEDOT:PSS (HTL) [53,54,55,56]
Thickness (µm)	0.05	0.4	0.05
$\mathrm{Bandgap}\;E_{g}$(eV)	1.7	1.4	1.8
Electron Affinity χ (eV)	4.50	3.670	3.40
Dielectric permittivity	18	8.2	18
CB effective density of states $\left({cm}^{-3}\right)$	$2.2\times 10^{18}$	$2.2\times 10^{18}$	$2.2\times 10^{18}$
VB effective density of states $\left({cm}^{-3}\right)$	$1.8\times 10^{19}$	$1.8\times 10^{19}$	$1.8\times 10^{19}$
Electron mobility $\left({cm}^{2}/V.s\right)$	$8\times 10^{-2}$	2	$4.5\times 10^{-2}$
Hole mobility $\left({cm}^{2}/V.s\right)$	$8\times 10^{-2}$	2	$4.5\times 10^{-2}$
Donor Concentration $N_{D}\;({cm}^{-3})$	$1\times 10^{17}$	$1\times 10^{13}$	$1\times 10^{7}$
Acceptor concentration $N_{A}\;({cm}^{-3})$	0	0	$1\times 10^{18}$

**Table 2:** Electrical and optical properties of different ETL materials.

Parameters	PCBM [53,54,55,56]	${TiO}_{2}$ [42,58]	ZnO [59,60]	${Zn}_{0.7}{Mg}_{0.3}O$ [56,61,62]
Thickness (µm)	0.05	0.05	0.05	0.05
Bandgap $E_{g}$ (eV)	2	3.26	3.3	4
Electron Affinity χ (eV)	4	4	3.7	3.65
Dielectric permittivity	3.9	32	9	8
CB effective density of states $\left({cm}^{-3}\right)$	$2.5\times 10^{21}$	$2.2\times 10^{18}$	$2.2\times 10^{18}$	$2.2\times 10^{18}$
VB effective density of states $\left({cm}^{-3}\right)$	$2.5\times 10^{21}$	$1.8\times 10^{19}$	$1.8\times 10^{19}$	$1.8\times 10^{19}$
Electron mobility $\left({cm}^{2}/V.s\right)$	$2.5\times 10^{-2}$	20	100	100
Hole mobility $\left({cm}^{2}/V.s\right)$	$2.5\times 10^{-2}$	10	25	25
Donor Concentration $N_{D}\;({cm}^{-3})$	$1\times 10^{17}$	$1\times 10^{17}$	$1\times 10^{17}$	$1\times 10^{17}$
Acceptor concentration $N_{A}\;({cm}^{-3})$	0	0	0	0

**Table 3:** Defect density values inside the layers and at the interface of the cell.

Parameters	ETL	HTL	Perovskite	HTL/Perovskite	Perovskite/ETL
Defect Type	Neutral	Neutral	Neutral	Neutral	Neutral
Capture cross-section for electrons $\sigma_{n}\;({cm}^{-2})$	$1\times 10^{-15}$	$1\times 10^{-15}$	$1\times 10^{-15}$	$1\times 10^{-18}$	$1\times 10^{-15}$
Capture cross-section for hole $\sigma_{p}\;({cm}^{-2})$	$1\times 10^{-15}$	$1\times 10^{-15}$	$1\times 10^{-15}$	$1\times 10^{-16}$	$1\times 10^{-15}$
Energetic distribution	Single	Single	Gaussian	Single	Single
Energy level with respect to $E_{v}$ (above $E_{v}$) (eV)	0.6	0.650	0.6	0.6	0.6
Characteristic energy (eV)	0.1	0.1	0.1	0.1	0.1
Total density $N_{t}\;({cm}^{-3})$	$1\times 10^{15}$	$1\times 10^{15}$	$1\times 10^{16}$	$1\times 10^{12}$	$1\times 10^{12}$

**Table 4:** Conduction band offset for the ETL materials and their photovoltaic properties.

ETL	CBO/eV	PCE/%	$V_{oc}/V$	$J_{sc}/mA\cdot {cm}^{-3}$	FF/%
C60	−0.83	8.47	0.51	21.97	75.43
PCBM	−0.33	11.64	0.60	24.64	78.09
$TiO_{2}$	−0.33	14.43	0.65	27.61	80.02
ZnO	−0.02	20.88	0.95	27.75	78.61
${Zn}_{0.7}{Mg}_{0.3}O$	0.02	21.55	0.94	27.86	82.33

**Table 5:** Conduction band offset for the ETL materials and their photovoltaic properties.

Metal	Al	Ag	Cr	Au	Ni	Pd	Pt
work function $\emptyset_{M}/eV$	4.125	4.26	4.4	5.1	5.15	5.3	5.15

## Data Availability

Not applicable.
